# Effect of diode laser combined with minocycline hydrochloride in nonsurgical periodontal therapy: a randomized clinical trial

**DOI:** 10.1186/s12903-022-02106-4

**Published:** 2022-03-14

**Authors:** Congchong Yang, Xikai Wang, Yanli Wang

**Affiliations:** 1 Department of Cariology and Endodontology, Shanghai Ninth People’s Hospital, Shanghai Jiao Tong University School of Medicine, Shanghai, China; 2 College of Stomatology, Shanghai Jiao Tong University, Shanghai, China; 3 National Center for Stomatology, National Clinical Research Center for Oral Diseases, Shanghai Key Laboratory of Stomatology, Shanghai, China; 4 Department of Occupational Disease, Xi’an Central Hospital, Xi’an, Shaanxi China; 5 Department of Stomatology, Xi’an Central Hospital, No. 161 Xiwu Road, Xincheng District, Xi’an, 710003 Shaanxi China

**Keywords:** Diode laser, Minocycline hydrochloride, Scaling and root planning, Nonsurgical periodontal therapy

## Abstract

**Background:**

This study aimed to assess the effect of diode laser combined with minocycline hydrochloride in conventional nonsurgical periodontal therapy.

**Methods:**

Ninety-two patients and 1206 teeth were included in this study. The patients were diagnosed moderate or severe periodontal diseases with the presence of teeth in at least 3 quadrants in the oral cavity. Each patient’s quadrants were randomly divided into three treatment groups as following, Control group: scaling and root planning (SRP); Experimental group 1 (Exp 1): SRP + minocycline hydrochloride; Experimental group 2 (Exp 2): SRP + 809 nm diode laser + minocycline hydrochloride. The minocycline in Exp 1 and Exp 2 was applied once per week, for 4 weeks. Clinical examinations including periodontal probing depth (PD), clinical attachment level (CAL) and bleeding index (BI), and the secretion of inflammatory factor (tumor necrosis factor, TNF-α) was detected by ELISA before and 3, 6 months after the treatments. The differences among these groups were assessed by One-Way ANOVA and Kruskal–Wallis test. *P*-value < 0.05 was considered significant.

**Results:**

All the periodontal indexes (PD, CAL and BI) were improved after each treatment and the secretion of TNF-α was reduced for all three groups. In patients with deep periodontal pockets, Exp 2 showed significant improvements in all indexes comparison with Con group and Exp group 1.

**Conclusions:**

The synergistic effect of SRP and 809 nm diode laser combined with minocycline hydrochloride could play an efficient and reliable effect in the nonsurgical periodontal treatment approach.

Trial registration The clinical trial was retrospectively registered in chictr.org.cn with registration ChiCTR2100051708 (01/10/2021).

## Background

Periodontitis is a chronic inflammatory disease which results in the destruction of periodontal connective tissue, alveolar bone resorption, and tooth loss [1, 2]. It is regarded as a major public health problem contributing to the global burden of chronic diseases with high prevalence worldwide [3].

Scaling and root planning (SRP) is purposed of removing the bacterial biofilm from root surfaces, which is the major clinical therapeutic method for periodontitis [4]. Although SRP could reduce numerous periodontal pathogens, the disease was still prone to relapses because of the ability of periodontopathic bacteria to penetrate and settle into gingival epithelial cells, cementum and radicular dentin [5, 6]. Various therapies are reported to apply to improve the effects of SRP, such as surgery (sub-gingival curettage, open flap debridement, osseous surgery) and antibacterial agents. However, during the surgical procedures, the clinical studies indicated that patients with the deepest of the periodontal pockets exposed the highest risk of losing the key clinical severity markers of periodontitis, including probing depth (PD) and clinical attachment level (CAL) [7]. Moreover, Hung et al. [8] demonstrated that flap debridement in deep periodontal pockets resulted in immediate pocket reduction compared with SRP alone, but the differences disappeared after 6 months.

Minocycline hydrochloride, a broad-spectrum antibiotic which was found to have a potential anti-inflammatory efficacy in periodontitis. It has also been proved to play a role in the immune response caused by cytokines and has beneficial effect on various indicators of periodontal health, and the infected localized areas were suitable for treatment with antimicrobial agents [9]. However, long-term use of the broad-spectrum antibiotic may increase the risk of side effects and the possibility of microbial resistance [10].

In recent years, it is expected that the use of laser could be served as an alternative or auxiliary treatment to conventional and mechanical periodontal therapy. Laser has various advantageous characteristics, such as haemostatic effects, selective calculus ablation, or bactericidal effects on periodontopathic pathogens, which may ultimately improve the treatment outcomes [11–13]. The most commonly used wavelengths of laser for periodontitis including semiconductor diode lasers, the Nd:YAG laser (Neodymium Doped: Yttrium, Aluminium, and Garnet), the Er:YAG laser (Erbium Doped: Yttrium, Aluminium, and Garnet), and the carbon dioxide (CO_2_) laser, range from 635 to 10,600 nm [14]. Previous studies have shown that diode laser and Nd:YAG laser are mainly used for laser-assisted subgingival curettage and periodontal pocket disinfection, with varying degrees of success [15, 16]. Clinical research has demonstrated limited or no clinical advantages, especially when lasers were used in the place of SRP [17]. However, studies specially focused on the effects of laser combined with SRP and antibiotic are limited.

Herein, diode laser is used combined with minocycline hydrochloride in conventional nonsurgical periodontal therapy in this study. Our specific objective is to compare the efficacy of SRP, SRP + minocycline hydrochloride, and SRP + diode laser + minocycline hydrochloride in the treatment of chronic periodontitis, and to further identify the synergistic effect of diode laser treatment with traditional periodontal treatment in patients with deep periodontal pockets.

## Methods

The study retrospectively registered in chictr.org.cn with registration ChiCTR2100051708 (01/10/2021). It was approved by the Ethical Committee of the Xi’an Central Hospital (2021-019). The study was conducted as a randomized controlled trial design. Participating subjects read and signed the informed consent prior to enrolling in the study. All methods were performed in accordance with the relevant guidelines and regulations.

### Sample selection

This study was conducted on the patients of clinically diagnosed periodontal disease in the Department of Stomatology of Xi’an Central Hospital between May 2018 and September 2020. The sample size of our study was calculated after conducting a statistical analysis. All patients were selected according to strict inclusion and exclusion criteria and signed a written informed consent document before treatment.

The inclusion criteria were as follows: (1) at least 16 natural teeth present in the oral distributed in 4 quadrants; (2) Moderate to severe periodontal disease with presence of 3 or more quadrants in the oral cavity, each containing at least three teeth (excluding third molar, supernumerary tooth or minor tooth) with periodontal pocket depth (PD) of ≥ 4 mm; (3) male or female individuals 20 to 60 years of age could exactly follow the investigator’s arrangement and sign the informed consent form voluntarily; (4) Light to moderate smokers (< 10 cigarettes/day) corresponding to a significant percentage of patients in our daily practice.

Exclusion criteria were as follows: (1) System diseases that affect the periodontal disease, such as diabetes, acquired immunodeficiency syndrome; (2) Patients presenting with known adverse reactions to any component of the test agent; (3) Taking systemic nonsteroidal anti-inflammatory drugs or immunomodulatory agents within the past 6 months; (4) Pregnancy or lactation or non-cooperator.

Each patient’s quadrant was randomly divided into three treatment groups as follows: Control group received only mechanical therapy (SRP); Experimental group 1 received SRP + minocycline hydrochloride; Experimental group 2 received SRP + diode laser + minocycline hydrochloride. The remaining quadrant was treated with SRP. The test sites in a patient were treated with different treatment procedures to compare their effects within the same individual.

### Treatment protocol

Every patient received oral hygiene instructions that included modified BASS brushing technique and mouth rinse without alcohol and chlorhexidine gluconate, twice a day after teeth brushing. Baseline examination including PD, CAL and bleeding index (BI) were performed 1 week before the experiment treatment. A week later, in the control group (Con), SRP was performed using manual Gracey curettes (Hu-Friedy, Chicago, USA) and ultrasonic scaler (EMS®, Switzerland). In the experimental group 1 (Exp 1), SRP was performed in the same manner as in Con group, followed by 10 mg minocycline hydrochloride (Sunstar, Japan) placed in periodontal pocket, once per week, for 4 weeks. In the experimental group 2 (Exp 2), the additional therapy was performed using Pilot laser (CAO Group, American) (tip diameter is 400 μm) 809 nm wavelength, 1.5 W power average output, operated in continuous mode. The laser probe paralleled to the root surface was gently inserted into the periodontal pocket, and moved evenly from bottom to coronally direction in order to covering the whole periodontal pocket. If the periodontal pocket depth < 6 mm, irradiation time was 30 s for one site; If the periodontal pocket depth > 6 mm, irradiation time was 45 s for one site. After irradiation, the periodontal pocket was alternately rinsed with 3% hydrogen peroxide and normal saline followed by minocycline hydrochloride as Exp 1.

### Clinical recordings

The periodontal status of each patient was assessed at baseline and at 3 and 6 months after treatment. All the measurements were done by one dentist who underwent calibration training at the beginning of the study and three repeated measurements were performed and had to show a > 90% agreement for -1 mm between initial and repeated probes. Thereby allowing intra-experimental comparisons of the values.

The following clinical and biological observations were recorded:

1. PD: by applying a calibrated periodontal probe (Hu-Friedy®, PQW7, USA) with a diameter of 0.5 mm, pocket depth was measured at 6 sites at each tooth (mesio-labial, mid-labial, distal-labial and mesio-palatal/lingual, mid-palatal/lingual and distal-palatal/lingual sites) as the distance from the gingival margin to the bottom of the pocket. The deepest PD from each tooth was selected as the test site.
2. CAL: this was recorded at 6 sites in a manner similar to PD in relation to the cementoenamel junction. The deepest CAL from each tooth was selected as the test site.
3. BI: using the same probing pressure, pocket bleeding was determined 30 s after probing and was assessed at 6 sites per tooth: 0- Healthy, no inflammation and bleeding; 1—Gingival inflammation changes, but no bleeding on probing; 2—Punctate bleeding; 3—Probing bleeding spreading along the gingival margin; 4—Bleeding over gingival sulcus; 5—Automatic bleeding.
4. Secretion of inflammatory factor (TNF-α): After drying the surface of all target teeth, a perio paper (Harco, Tuston, CA, USA) was taken and zeroed in Periotron 8000 (OraflowCompany, USA), and then inserted into the gingival sulcus at 4 sites at each tooth (mesio-labial, distal-labial, mesio-palatal/lingual and distal-palatal/lingual) for 30 s. The filter paper was transfered to EP tube filled with 500 μL phosphate buffer (PBS) for 4 °C overnight, centrifuged at 1500 r/min for 4 min and then get the supernatant [18]. TNF-α was measured by enzyme-linked immunosorbent assay (ELISA).

### Statistical analysis

Statistical analysis was performed using SPSS19.0 software (SPSS, Chicago, USA). All data were expressed as mean ± standard deviation (s.d.). All the data were normally distributed. The results of the parameters (PD, CAL, and TNF-α) were compared using the One-Way ANOVA among the groups at each time point. The Kruskal–Wallis test was applied to find the difference in the BI with each group at each time point. *P*-value < 0.05 was considered significant.

## Results

**Fig. 1:**
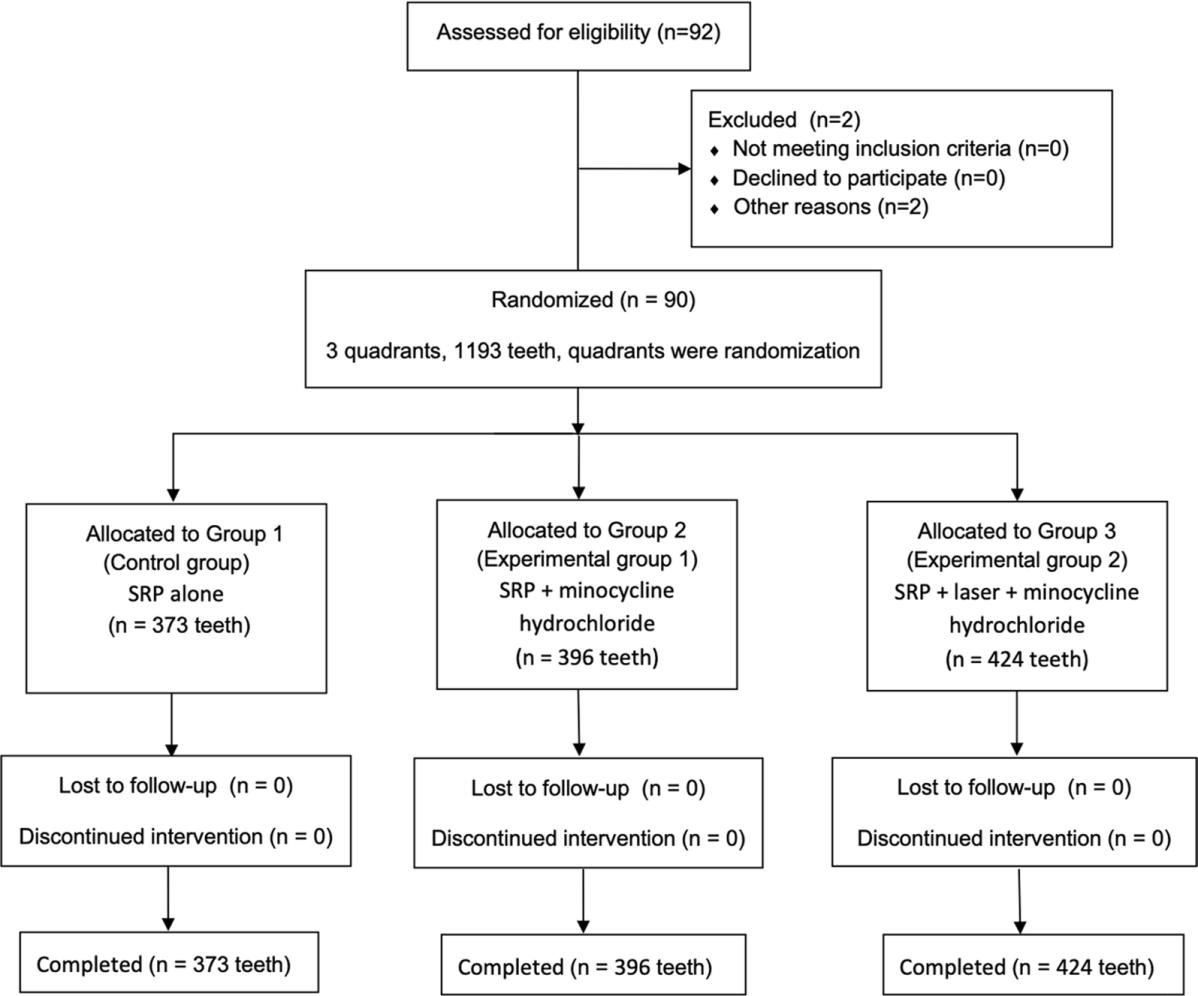
Flow chart of the study

As Fig. 1 shows, 92 patients were enrolled in this study. Two patients were not completed in this study because of personal work reasons. A total of 90 patients and 1193 teeth participated until the end. The mean age of the population was 47.3 ± 7.6 years, and almost two-thirds of the patients were male (59 men). 373 teeth in the control group, 396 teeth in the Exp 1, and 424 teeth in the Exp 2.

Two categories were assessed for the outcomes of treatment: site with baseline probing depth for moderately deep pockets (4 mm < PD < 6 mm) and deep pockets (PD > 6 mm). The three groups showed similar baseline clinical characteristics for the PD, CAL, BI and the secretion of TNF-α. Compared with baseline, all treatment groups were significant decreased at 3 months and 6 months.

### Clinical observations

PD: As showed in Table 1, the Exp 2 in the two periodontal pocket depths were significant improvement at 6 months compared with 3 months (the moderately deep pockets, *p* = 0.012; the deep pocket, *p* = 0.011) while there was no difference in the two groups else. Moreover, for the moderately deep pockets (4 mm < PD < 6 mm), the PD in the Exp 2 was significant lower than Con group (*p* < 0.001) and Exp1 (*p* = 0.001) at 6 months; For the deep pockets (PD > 6 mm), the Exp 1 and Exp 2 were significant lower than Con group at 3 months and 6 months (Exp 1, *p* < 0.001; Exp 2, *p* < 0.001), and the Exp 2 was lower than Exp 1 at 6 months (*p* = 0.042).

**Table 1:** Difference in PD parameter for moderately deep pockets (4 mm < PD < 6 mm) and deep pockets (PD > 6 mm)

PD	n (teeth)	Baseline	3 months	*p*_(0–3)_	6 months	*p*_(0–6)_	*p*_(3–6)_
*Moderately deep pockets (4 mm* < *PD* < *6 mm)*							
Con	152	4.93 ± 0.53	3.78 ± 0.53	< 0.001	3.79 ± 0.45	< 0.001	0.98
Exp 1	209	5.01 ± 0.46	3.64 ± 0.67	< 0.001	3.61 ± 0.37	< 0.001	0.819
Exp 2	211	4.96 ± 0.48	3.55 ± 0.49	< 0.001	3.25 ± 0.38	< 0.001	0.012
*p*		0.816	0.225		< 0.001** 0.001^△^		
*Deep pockets (PD* > *6 mm)*							
Con	221	7.37 ± 1.02	5.02 ± 0.69	< 0.001	4.79 ± 1.24	< 0.001	0.132
Exp 1	187	7.4 ± 0.13	3.91 ± 0.65	< 0.001	3.82 ± 0.95	< 0.001	0.585
Exp 2	213	7.33 ± 0.98	4 ± 1.02	< 0.001	3.52 ± 0.97	< 0.001	0.011
*p*		0.897	< 0.001*		< 0.001*		
			< 0.001**		< 0.001** 0.042^△^		

*p* value refers to the statistically significant difference between groups in the same period (*p* < 0.05); *p*_(0–3)_ value refers to the statistically significant difference for each group between baseline and 3 months (*p* < 0.05); *p*_(0–6)_ value refers to the statistically significant difference for each group between baseline and 6 months (*p* < 0.05); *p*_(3–6)_ value refers to the statistically significant difference for each group between 3 and 6 months (*p* < 0.05); *means difference between Con and Exp group 1; **means difference between Con and Exp group 2; ^△^ means difference between Exp 1 and Exp 2 groups

CAL: As showed in Table 2, compared with 3 months, the Exp 1 in the moderately deep pocket was decreased (*p* = 0.003) and the Exp 2 in the two periodontal pocket depths were improvement at 6 months (the moderately deep pockets, *p* = 0.001; the deep pocket, *p* = 0.006). For the deep pockets (PD > 6 mm), the CAL of Exp 2 was significant decreased than Exp 1 at 6 months (*p* = 0.028), while there was no difference in the three groups else (Table 2).

**Table 2:** Difference in CAL parameter for moderately deep pockets (4 mm < PD < 6 mm) and deep pockets (PD > 6 mm)

CAL	n (teeth)	Baseline	3 months	*p*_(0–3)_	6 months	*p*_(0–6)_	*p*_(3–6)_
*Moderately deep pockets (4 mm* < *PD* < *6 mm)*							
Con	152	5.19 ± 0.45	4.45 ± 0.45	< 0.001	4.28 ± 0.41	< 0.001	0.151
Exp 1	209	5.08 ± 1.21	4.38 ± 0.78	< 0.001	4.11 ± 0.51	< 0.001	0.003
Exp 2	211	5.13 ± 0.97	4.45 ± 1.09	< 0.001	4.08 ± 0.44	< 0.001	0.001
*p*		0.711	0.821		0.175		
*Deep pockets (PD* > *6 mm)*							
Con	221	7.47 ± 1.13	6.42 ± 0.85	< 0.001	6.10 ± 0.7	< 0.001	0.124
Exp 1	187	7.49 ± 0.7	6.74 ± 0.96	< 0.001	6.46 ± 0.79	< 0.001	0.188
Exp 2	213	7.5 ± 1.09	6.60 ± 1.0	< 0.001	5.99 ± 0.94	< 0.001	0.006
*p*		0.991	0.375		0.028^△^		

*p* value refers to the statistically significant difference between groups in the same period (*p* < 0.05); *p*_(0–3)_ value refers to the statistically significant difference for each group between baseline and 3 months (*p* < 0.05); *p*_(0–6)_ value refers to the statistically significant difference for each group between baseline and 6 months (*p* < 0.05);

*p*_(3–6)_ value refers to the statistically significant difference for each group between 3 and 6 months (*p* < 0.05); *p* value refers to the statistically significant difference between groups in the same period (*p* < 0.05); ^△^ means difference between Exp 1 and Exp 2 groups

BI: the Exp 2 in deep pocket was improvement at 6 months compared with 3 month (*p* = 0.043). No significant difference was observed in other groups at each time. For the deep pockets (PD > 6 mm), the BI of Exp 2 was significant decreased than Con group and Exp 1 at both 3 and 6 months (3 months, *p* = 0.013, *p* = 0.012; 6 months, *p* < 0.001, *p* < 0.001), while there were no difference in the moderately deep pockets (Table 3).

**Table 3:** Difference in BI parameter for moderately deep pockets (4 mm < PD < 6 mm) and deep pockets (PD > 6 mm)

BI	n (teeth)	Baseline	3 months	*p*_(0–3)_	6 months	*p*_(0–6)_	*p*_(3–6)_
*Moderately deep pockets (4 mm* < *PD* < *6 mm)*							
Con	152	4.33 ± 0.53	2.90 ± 0.70	0.023	2.88 ± 0.43	0.02	0.978
Exp 1	209	4.48 ± 0.96	2.33 ± 0.86	0.015	2.45 ± 0.73	0.019	0.833
Exp 2	211	4.29 ± 0.5	2.35 ± 0.64	0.013	2.42 ± 0.96	0.015	0.896
*p*		0.925	0.882		0.791		
*Deep pockets (PD* > *6 mm)*							
Con	221	4.57 ± 0.84	3.31 ± 0.66	0.037	3.37 ± 0.29	0.039	0.969
Exp 1	187	4.49 ± 0.91	3.4 ± 0.32	0.049	3.38 ± 0.72	0.049	0.972
Exp 2	213	4.47 ± 0.73	2.39 ± 0.48	< 0.001	2.01 ± 0.43	< 0.001	0.043
*p*		0.994	0.013** 0.012^△^		< 0.001** < 0.001^△^		

*p* value refers to the statistically significant difference between groups in the same period (*p* < 0.05); *p*_(0–3)_ value refers to the statistically significant difference for each group between baseline and 3 months (*p* < 0.05); *p*_(0–6)_ value refers to the statistically significant difference for each group between baseline and 6 months (*p* < 0.05); *p*_(3–6)_ value refers to the statistically significant difference for each group between 3 and 6 months (*p* < 0.05); **means difference between Con and Exp 2 groups; ^△^ means difference between Exp 1 and Exp 2 groups

### Biological observations

TNF-α: As showed in Table 4, compared with 3 months, the Exp 1 in the deep pocket was decreased (*p* = 0.044) and the Exp 2 in the two periodontal pocket depths were improvement at 6 months (the moderately deep pockets, *p* = 0.037; the deep pocket, *p* = 0.017). For the moderately deep pockets (4 mm < PD < 6 mm), the TNF-α of Exp 2 was lower than Con group and Exp 1 at 6 months (Con group 1, *p* = 0.002; Exp 1, *p* = 0.035). For the deep pockets (PD > 6 mm), the TNF-α of Exp 2 was lower than Con group at 3 months (*p* = 0.007) and lower than Con group and Exp 2 at 6 months (Con group 1, *p* < 0.001; Exp 1, *p* = 0.002) (Table 4).

**Table 4:** Difference in TNF-α parameter for moderately deep pockets (4 mm < PD < 6 mm) and deep pockets (PD > 6 mm)

TNF-α (μg/L)	n (teeth)	Baseline	3 months	*p* (0–3)	6 months	*p* (0–6)	*p* (3–6)
*Moderately deep pockets (4 mm* < *PD* < *6 mm)*							
Con	152	6.51 ± 1.29	4.73 ± 1.20	< 0.001	4.60 ± 1.11	< 0.001	0.683
Exp 1	209	6.40 ± 1.20	4.51 ± 1.22	< 0.001	4.36 ± 0.91	< 0.001	0.606
Exp 2	211	6.49 ± 1.29	4.43 ± 1.22	< 0.001	3.84 ± 0.84	< 0.001	0.037
*p*		0.943	0.605		0.002** 0.035^△^		
*Deep pockets (PD* > *6 mm)*							
Con	221	7.12 ± 1.47	5.42 ± 1.15	< 0.001	5.03 ± 0.99	< 0.001	0.217
Exp 1	187	7.05 ± 1.43	5.22 ± 0.97	< 0.001	4.64 ± 0.78	< 0.001	0.044
Exp 2	213	7.22 ± 1.61	4.67 ± 1.09	< 0.001	3.94 ± 0.79	< 0.001	0.017
*p*		0.908	0.007**		< 0.001** 0.002^△^		

*p* value refers to the statistically significant difference between groups in the same period (*p* < 0.05); *p* (0–3) value refers to the statistically significant difference for each group between baseline and 3 months (*p* < 0.05); *p* (0–6) value refers to the statistically significant difference for each group between baseline and 6 months (*p* < 0.05); *p* (3–6) value refers to the statistically significant difference for each group between 3 and 6 months (*p* < 0.05); *p* value refers to the statistically significant difference between groups in the same period (*p* < 0.05); **means difference between Con and Exp 2 groups; △ means difference between Exp 1 and Exp 2 groups

## Discussion

Periodontitis is a chronic inflammatory disease caused by pathogenic microflora in the biofilm [19]. The main purpose of periodontal therapy is to remove the calculus and inflamed cementum and arrest the inflammatory disease response [20], which can be carried out by non-surgical or surgical methods. Haffajee et al. [21]. have shown that levels of Tannerella forsythia, Fusobacterium nucleatum, Porphyromonas gingivalis, and Treponema denticola decreased significantly until 3 months after subgingival debridement. Cleland et al. [22] have also stated that bacterial re-colonization of subgingival plaque occurs in deep pockets within 120–240 days. Therefore, in this study, the observation period of the periodontal examination was 3 months and 6 months, respectively, in order to eliminate any potential risks that may influence the bactericidal outcomes related to the speed and degree of further biofilm re-colonization. Each patient quadrant was allocated to one of the three treatment groups randomly, in order to eliminate individual differences.

The clinical effect of minocycline hydrochloride is that at the site of infection, bacteria are exposed to higher doses of antibiotics, reducing the risk of resistant bacterial strains and reaching a concentration higher than the minimum inhibitory concentration of putative pathogens [23]. Minocycline hydrochloride have been proved to exhibit anti-inflammatory activity by inhibiting macrophages [24]. Moreover, Cortelli et al. [25] evaluated that minocycline hydrochloride in the treatment of chronic periodontitis had statistically significant improvement in the gingival index scores at 6 weeks and 3 months.

Lasers is mainly used for periodontal soft tissue surgery, and is suitable for tissue ablation, hemostasis and disinfection in photoablative mode. Kreisler et al. [26] indicated that the adjunctive use of 809 nm diode laser radiation might have a positive influence on wound healing following SRP. To date, there is no report evaluating the 809 nm diode laser combined with minocycline hydrochloride in conjunction with SRP procedure for treatment of periodontal disease.

Therefore, in our study, the PD, CAL, BI and the secretion of TNF-α in the gingival crevicular fluid showed a similar reduction in all three groups at 3 and 6 months compared with the baseline after treatment. In the Exp 2, a tendency for reduction in these clinical indexes at 6 months compared with 3 months except for BI for the moderately deep pockets (4 mm < PD < 6 mm), while there was no significant difference in the Con group and Exp group 1 between 3 and 6 months except the PD and TNF-α in Exp 1 for the moderately deep pockets (4 mm < PD < 6 mm). For the moderately deep pockets (4 mm < PD < 6 mm), there was no difference of PD among the three groups at 3 months, but a tendency for greater PD of Exp 2 reduction was observed at 6 months. For the deep pockets (PD > 6 mm), the PD in Exp 1 was lower than Con group at 3 and 6 months, that of Exp 2 was lower than Con group at 3 months and lower than Con group and Exp 1 at 6 months.

Many studies on periodontitis suggested that minocycline hydrochloride [27] or laser [28] could improve the CAL of patients. However, the present results showed there was no statistically significant difference among groups at 3 and 6 months except for the CAL in the Exp 2 in comparison with the Exp 1 for deep pockets (PD > 6 mm) at 6 months. This result indicates that the depth of periodontal pocket is an important factor affecting the therapeutic effect.

Concerning the BI, the moderately deep pockets (4 mm < PD < 6 mm) had no difference among groups, while for the deep pockets (PD > 6 mm), the index in the Exp 2 decreased greater than that of the Con group and Exp 1. It must also be emphasized that this specific wavelength is well absorbed in haemoglobin in deep periodontal pocket.

For the moderately deep pockets (4 mm < PD < 6 mm), the secretion of TNF-α in Exp 2 was lower than Con group and Exp 1 at 6 months. For the deep pockets (PD > 6 mm), the secretion of TNF-α was greater decreased in the Exp 2 than Con group both at 3 and 6 months, and lower than Exp 1 at 6 months. This result indicates that the effect of diode laser combined with minocycline hydrochloride in anti-inflammatory treatment is much better.

## Conclusions

In summary, our results showed that diode laser combined minocycline hydrochloride after SRP could improve the clinical periodontal indexes, such as PD, CAL and BI, and reduce the secretion of TNF-α especially in the deep pockets. In addition, the use of different laser parameters such as irradiate time and power should be further investigated. Collectively, the synergistic effect of SRP and 809 nm diode laser combined with minocycline hydrochloride could play an efficient and reliable effect in the nonsurgical periodontal treatment approach.

## Data Availability

The data and materials collected in this research are available from corresponding author when requested reasonably.
